# Association between serum chloride levels with mortality in critically ill patients with acute kidney injury: An observational multicenter study employing the eICU database

**DOI:** 10.1371/journal.pone.0273283

**Published:** 2022-08-23

**Authors:** Xu Zhu, Jing Xue, Zheng Liu, Wenjie Dai, Jingsha Xiang, Hui Xu, Qiaoling Zhou, Quan Zhou, Wenhang Chen

**Affiliations:** 1 Department of Epidemiology and Health Statistics, College of Integrated Traditional Chinese and Western Medicine, Hunan University of Chinese Medicine, Changsha, Hunan, China; 2 Department of Scientific Research, Xiangya Hospital, Central South University, Changsha, Hunan, China; 3 Department of Anesthesiology, Shandong Provincial Qianfoshan Hospital, The First Hospital Affiliated with Shandong First Medical University, Ji’nan, Shandong, China; 4 Xiangya School of Public Health, Central South University, Changsha, Hunan, China; 5 Department of Nephrology, Xiangya Hospital, Central South University, Changsha, Hunan, China; 6 Department of Science and Education, The First People’s Hospital of Changde City, Changde, Hunan, China; 7 Movement System Injury and Repair Research Center, Xiangya Hospital, Central South University, Changsha, Hunan, China; Federal Medical Centre Umuahia, NIGERIA

## Abstract

**Objective:**

The effect of the serum chloride (Cl) level on mortality in critically ill patients with acute kidney injury (AKI) remains unknown. We sought an association between mortality and serum Cl.

**Methods:**

We identified AKI patients in the eICU Collaborative Research Database from 2014 to 2015 at 208 US hospitals. The outcomes included in-hospital and intensive care unit (ICU) mortality. Time-varying covariates Cox regression models and the Kaplan-Meier (K-M) curves were used to assess the association between serum Cl levels and mortality. Multivariable adjusted restricted cubic spline models were used to analyze the potential nonlinear relationship between mortality and serum Cl.

**Results:**

In total, 4,234 AKI patients were included in the study. Compared with normochloremia (98≤chloride<108mEq/L), hypochloremia (Cl<98mEq/L) was associated with mortality (adjusted hazard ratio [HR] for in-hospital mortality 1.46, 95% confidence interval [CI] 1.20–1.80, P = 0.0003; adjusted HR for ICU mortality 1.37, 95% CI 1.05–1.80, P = 0.0187). Hyperchloremia showed no significant difference in mortality compared to normochloremia (adjusted HR for in-hospital mortality 0.89, 95% CI 0.76–1.04, P = 0.1438; adjusted HR for ICU mortality 0.87, 95% CI 0.72–1.06, P = 0.1712). Smoothing curves revealed continuous non-linear associations between serum Cl levels and mortality. The K-M curve showed that patients with hypochloremia presented with a lower survival rate.

**Conclusions:**

Lower serum Cl levels after ICU admission was associated with increased in-hospital and ICU mortality in critically ill patients with AKI. The results should be verified in well-designed prospective studies.

## Introduction

Acute kidney injury (AKI) is manifested by an elevated serum creatinine level and/or decreased urine output attributable to abrupt deterioration in kidney function [1]. AKI is associated with an increased risk of later chronic kidney disease (CKD), end-stage renal disease, cardiovascular events, and in-hospital and long-term mortality [2, 3]. Many patients, especially patients in intensive care units (ICUs), are at risk of AKI [4–6].

Chloride (Cl) is the principal extracellular anion in the human body; it contributes approximately one-third of all extracellular fluid tonicity. Serum Cl exerts many physiological functions including maintenance of the osmotic and acid–base balance, body fluid distribution, and muscular activity [7]. The kidneys are important regulators of Cl homeostasis. Renal tubular Cl re-absorption is critical in terms of extracellular fluid volume maintenance [8]. Dyschloremia (both hypochloremia and hyperchloremia) is common in critically ill patients; it is attributable to various etiological factors or treatments [7]. Dyschloremia has also been associated with worse outcomes among patients in ICUs or coronary care units [9–11]. Importantly, dyschloremia is an independent prognostic predictor of hypertensive patients [12], decompensated cirrhosis [13], chronic heart failure [14], and CKD [15], as well as pediatric patients [16]. The causal link between hyperchloremia and the risk of AKI has yet to be proven [17–19]. In critically ill patients, hypochloremia is associated with an increased risk of development of AKI [20]. However, the impact of the serum Cl level on clinical outcomes in patients with AKI remains poorly characterized. Here, we explored the association between the serum Cl level and the risk of mortality in critically ill AKI patients.

## Materials and methods

### Data source

This retrospective observational study used data from the eICU Collaborative Research Database (eICU-CRD) ver. 2.0, which constitutes a large, publicly available multicenter database regarding 200,859 ICU admissions of 139,367 patients from 2014 to 2015 at 208 US hospitals [21]. The eICU database is de-identified but contains comprehensive records, including demographics, physiological readings from bedside monitors, diagnoses, treatment information, and other clinical data collected during routine medical care. The use of the database was approved by the Institutional Review Board (IRB) of the Massachusetts Institute of Technology (Cambridge, MA, USA). Xu Zhu, an author of this study, completed the “Protecting Human Research Participants” curriculum and then accessed the eICU-CRD data (authorization code 41711250). The study was approved by the Institutional Review Board of the Xiangya Hospital of Central South University. All methods were carried out in accordance with relevant guidelines and regulations. The results are reported in accordance with the STrengthening the Reporting of OBservational studies in Epidemiology (STROBE) Statement [22].

### Selection criteria

Patients with acute renal failure were potentially eligible. The exclusion criteria were: not the first ICU admission in the database; ICU length of stay (LOS) < 24 h; age < 18 years; loss of > 5% of all data; missing serum Cl data within 72 h after ICU admission; missing LOS data, and the absence of survival outcomes including in-hospital or ICU mortality.

### Data collection

PostgreSQL (ver. 9.6) was used to extract all variables and outcomes in Structured Query Language (SQL) format. Demographic information included age, sex, ethnicity, weight and height on admission. Comorbidities included sepsis, CKD, hypertension, heart failure, coronary artery disease, diabetes, pneumonia. Laboratory parameters included the levels of sodium, potassium, magnesium, total calcium, phosphate, bicarbonate, albumin, creatinine, and blood urea nitrogen. Laboratory parameters were obtained within 72 h after ICU admission. The first laboratory value (except the value of Cl) was included if there were multiple values. The APACHE IV score was extracted to assess illness. In terms of treatments, the following data were extracted: use of antibiotics, diuretics, and vasopressors, as well as mechanical ventilation statuses. AKI was staged in accordance with the Kidney Disease: Improving Global Outcomes guidelines [23]. Survival statuses at hospital and ICU discharge were recorded.

### Endpoints

The primary outcome was all-cause in-hospital mortality (i.e., survival at hospital discharge). ICU mortality, defined as survival at ICU discharge, was considered a secondary outcome.

### Statistical analysis

Participants were groups as follows: normochloremia (98≤Cl<108mEq/L), hypochloremia (Cl<98mEq/L) and hyperchloremia (Cl≥108mEq/L). Normal serum Cl was used as the reference category. If variables were normally distributed and the variance was homogeneous, the data are expressed as means ± standard deviations; they were compared between groups using Student’s t-test. Otherwise, the data are presented as medians with interquartile ranges, and the Wilcoxon rank-sum test was used for between-group comparisons of such variables. Categorical variables are expressed as numbers with proportions; between-group comparisons of such variables were made using the chi-squared test or Fisher’s exact test (as appropriate). Time-varying covariates Cox regression models were used to estimate the hazard ratios (HRs) for associations between Cl levels and outcomes in both univariate and multivariate analyses [24]. Two multivariate models were constructed based on the adjusted variables. Model I adjusted for age, sex, and ethnicity; model II adjusted for variables in model I, along with weight and height on admission, comorbidities, laboratory data, and treatment. We explored the potential nonlinear relationship between serum Cl levels and mortality by using multivariable adjusted restricted cubic spline models [25]. Kaplan-Meier curves were plotted to calculate the cumulative survival rates according to serum Cl categories [26]. Their differences were analyzed by the log-rank test. A two-tailed p-value < 0.05 was considered statistically significant. All statistical analyses were performed using R software (ver. 4.1.2; R Foundation for Statistical Computing, Vienna, Austria) and Empower (R) (X & Y Solutions, Inc., Boston, MA, USA).

## Results

### Patient characteristics

**Fig 1:**
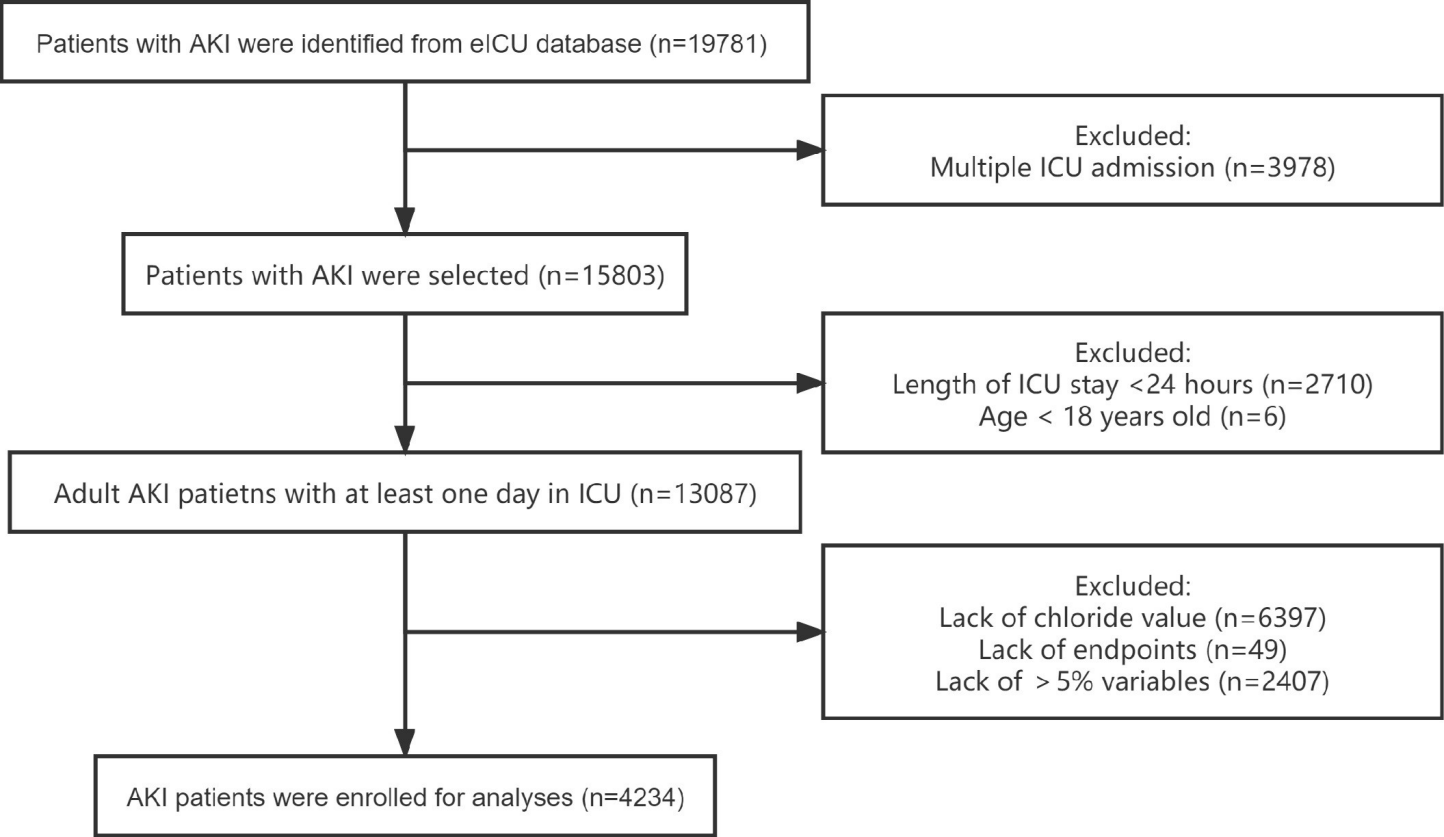
Flowchart of included patients. Abbreviations: AKI, acute kidney injury; ICU, intensive care unit.

Data regarding 19,781 AKI patients were initially obtained from the eICU database; of these patients, 4,234 were included in the analysis (Fig 1). Their general characteristics are presented in Table 1. The mean age was 65.78 years and 2,414 (57.01%) patients were male. Most patients were Caucasian (72.32%). The leading comorbidity was sepsis (38.14%). Of all patients, 48.82% required mechanical ventilation and 12.87% patients were treated with diuretics.

**Table 1:** Baseline clinical and laboratory characteristics of the study patients.

Variables	Total	Survival group	Death group	*P*-value
Number	4234	3228	1006	
Age (years)	65.78 ± 15.36	64.87 ± 15.66	68.71 ± 13.97	<0.001
Gender				0.404
Female, n (%)	1820 (42.99%)	1399 (43.34%)	421 (41.85%)	
Male, n (%)	2414 (57.01%)	1829 (56.66%)	585 (58.15%)	
Ethnicity				0.445
Caucasian, n (%)	3062 (72.32%)	2325 (72.03%)	737 (73.26%)	
Others, n (%)	1172 (27.68%)	903 (27.97%)	269 (26.74%)	
Admission weight (kg)	87.97 ± 28.54	88.28 ± 28.34	86.96 ± 29.17	0.199
Admission height (cm)	169.45 ± 13.41	169.50 ± 13.03	169.30 ± 14.57	0.689
Comorbidity				
Sepsis, n (%)	1615 (38.14%)	1170 (36.25%)	445 (44.23%)	<0.001
Diabetes, n (%)	595 (14.05%)	470 (14.56%)	125 (12.43%)	0.089
Pneumonia, n (%)	784 (18.52%)	554 (17.16%)	230 (22.86%)	<0.001
Heart failure, n (%)	601 (14.19%)	442 (13.69%)	159 (15.81%)	0.094
Hypertension, n (%)	624 (14.74%)	502 (15.55%)	122 (12.13%)	0.007
Coronary artery disease, n (%)	217 (5.13%)	163 (5.05%)	54 (5.37%)	0.689
CKD, n (%)	451 (10.65%)	334 (10.35%)	117 (11.63%)	0.249
Laboratory-based data				
Sodium (mmol/L)	138.27 ± 6.93	138.16 ± 6.84	138.64 ± 7.21	0.055
Potassium (mmol/L)	4.39 ± 0.95	4.40 ± 0.96	4.39 ± 0.91	0.776
Magnesium (mmol/L)	1.96 ± 0.49	1.96 ± 0.49	1.97 ± 0.49	0.517
Total calcium (mg/dL)	8.04 ± 1.01	8.06 ± 0.99	7.97 ± 1.07	0.009
Bicarbonate (mEq/L)	20.79 ± 5.71	20.98 ± 5.76	20.20 ± 5.51	<0.001
Phosphate (mmol/L)	4.41 ± 1.94	4.29 ± 1.88	4.79 ± 2.05	<0.001
Albumin (g/dL)	2.64 ± 0.61	2.67 ± 0.60	2.53 ± 0.64	<0.001
Creatinine (mg/dL)	2.20 (1.49–3.57)	2.24 (1.49–3.70)	2.10 (1.49–3.18)	<0.001
BUN (mg/dL)	44.00 (28.00–66.00)	44.00 (28.00–67.00)	42.50 (27.00–64.00)	0.120
Treatment information, n (%)				
Antibiotics, n (%)	1489 (35.17%)	1134 (35.13%)	355 (35.29%)	0.927
Vasopressors, n (%)	1879 (44.38%)	1183 (36.65%)	696 (69.18%)	<0.001
Diuretic, n (%)	545 (12.87%)	414 (12.83%)	131 (13.02%)	0.871
Mechanical ventilation, n (%)	2067 (48.82%)	1323 (40.99%)	744 (73.96%)	<0.001
APACHE IV score	79.97 ± 27.77	75.02 ± 24.67	95.86 ± 31.00	<0.001

Values are expressed as mean (standard deviation [SD]) or median (interquartile range [IQR]).

Abbreviations: APACHE, acute physiology and chronic health evaluation; BUN, blood urea nitrogen; CKD, chronic kidney disease.

### Associations between serum Cl and outcomes

**Fig 2:**
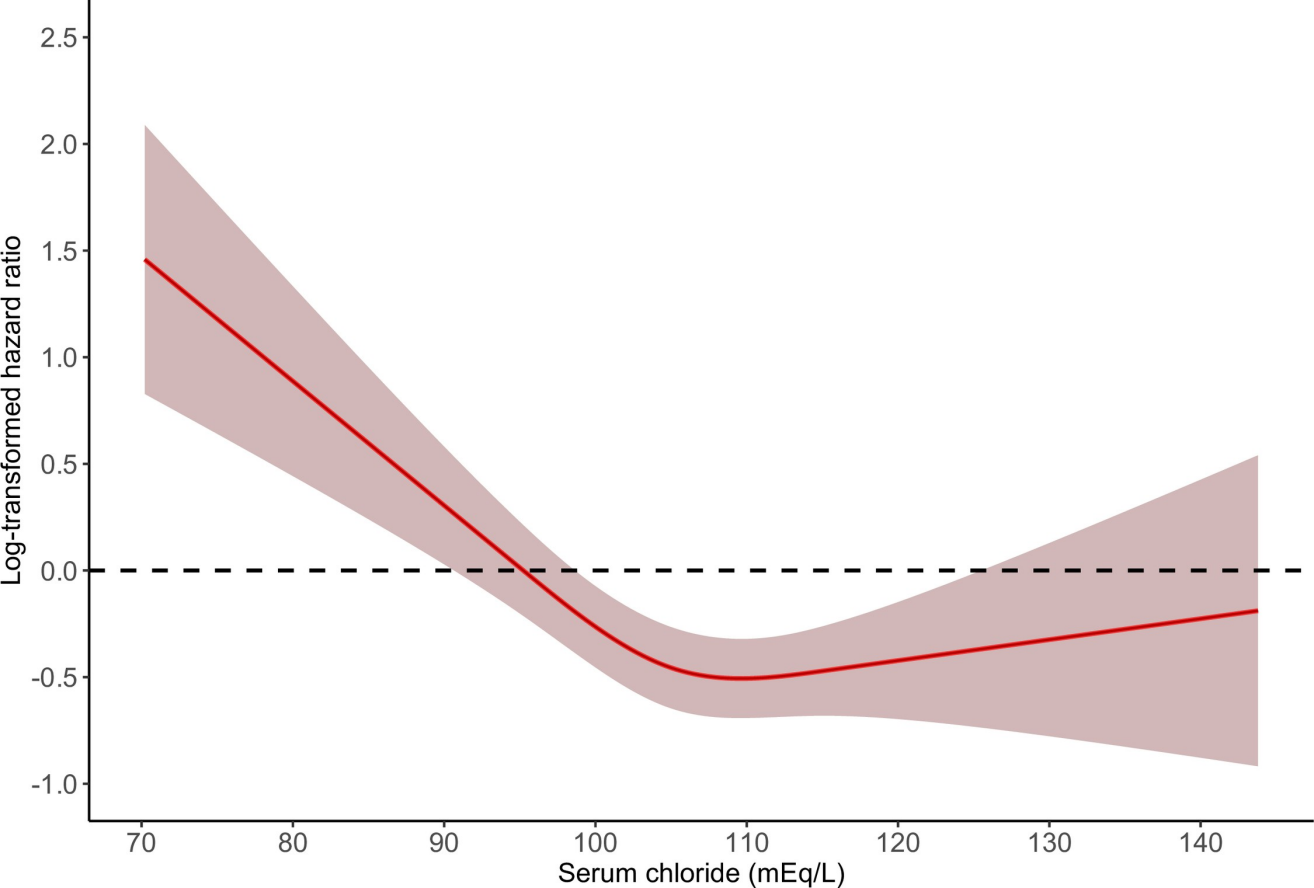
The smoothing curves of in-hospital mortality of critically ill AKI patients against serum chloride. The solid red lines represent the effect estimates and the shaded area represents 95% confidence intervals. *P* for non–linearity = 0.0001. Abbreviations: AKI, acute kidney injury.

**Fig 3:**
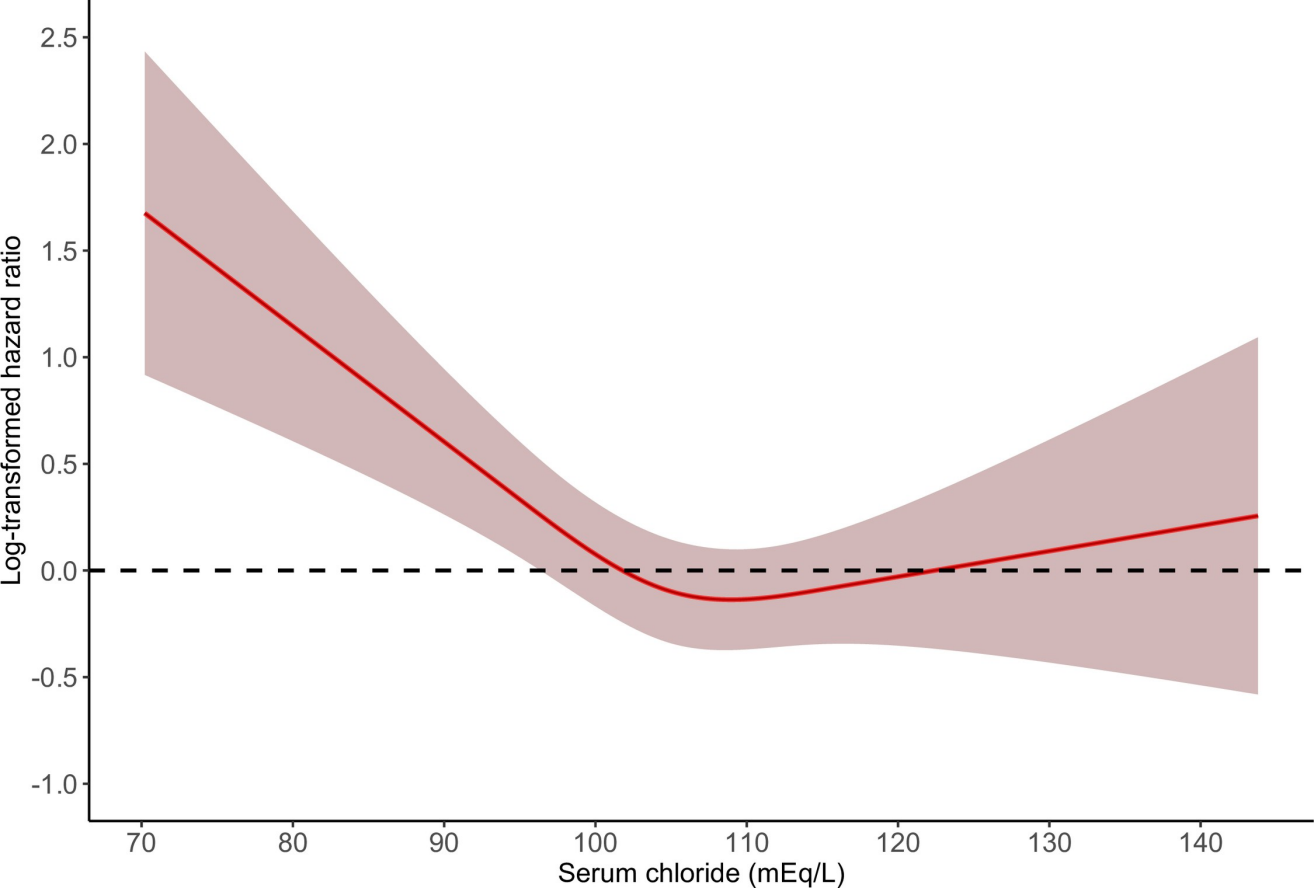
The smoothing curves of ICU mortality of critically ill AKI patients against serum chloride. The solid red lines represent the effect estimates and the shaded area represents 95% confidence intervals. *P* for non–linearity = 0.0015. Abbreviations: AKI, acute kidney injury; ICU, intensive care units.

**Fig 4:**
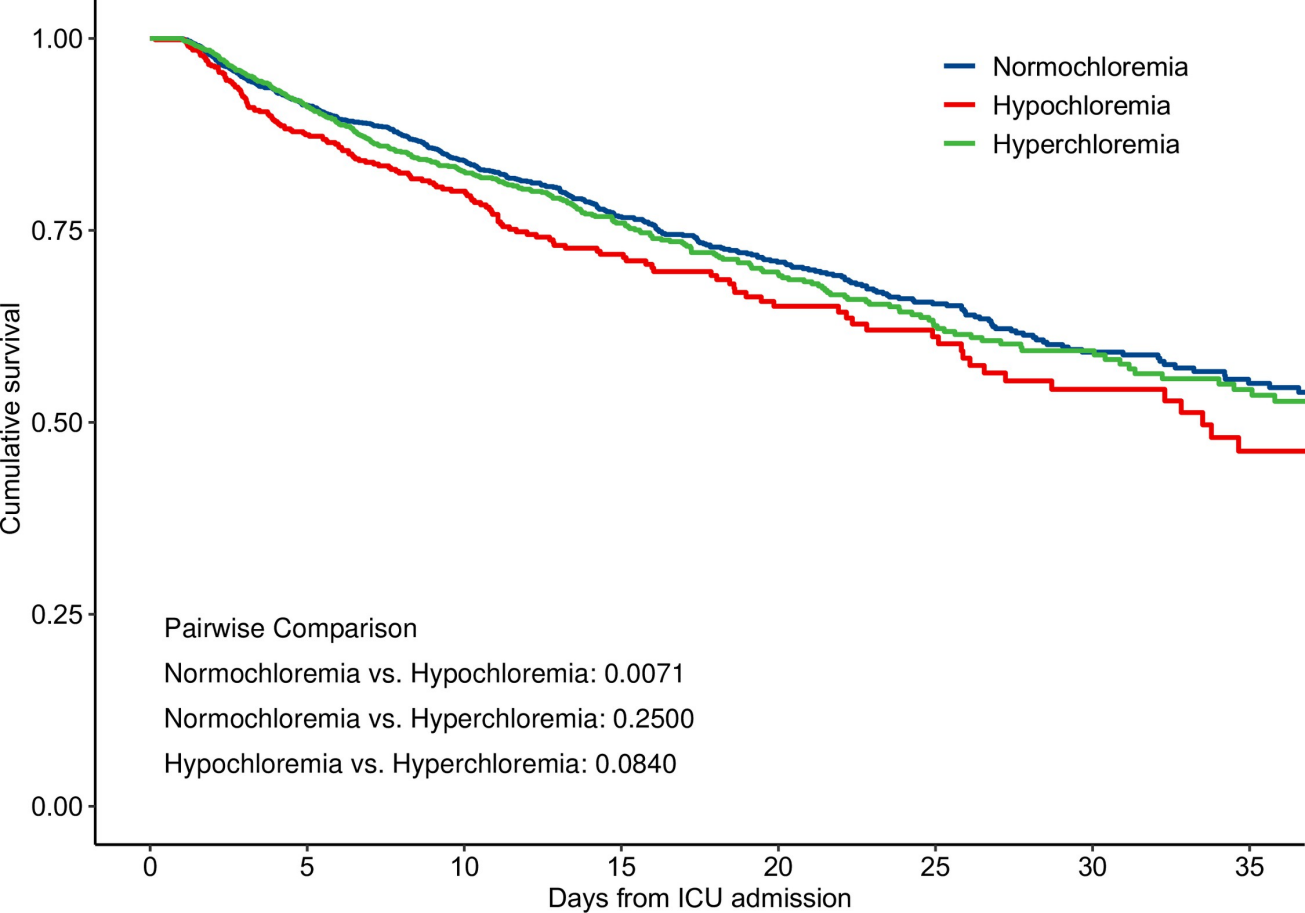
Kaplan-Meier survival curve for in-hospital mortality stratified by serum chloride in three groups. Log rank p-values between groups were reported.

In the time-varying covariates Cox regression modes, compared with normal serum Cl, hypochloremia had significantly higher risks of both in-hospital mortality and ICU mortality (adjusted HR 1.46, 95% CI 1.20–1.80, P = 0.0003; adjusted HR 1.37, 95% CI 1.05–1.80, P = 0.0187; respectively) (Table 2). Hyperchloremia was not significantly associated with in-hospital mortality and ICU mortality (adjusted HR 0.89, 95% CI 0.76–1.04, P = 0.1438; adjusted HR 0.87, 95% CI 0.72–1.06, P = 0.1712; respectively). As shown in Figs 2 and 3, we observed non-linear associations between serum Cl and in-hospital, ICU mortality (*P*_non–linearity_ for in-hospital mortality = 0.0001; *P*_non–linearity_ for ICU mortality = 0.0015). Fig 4 shows the Kaplan-Meier curve for in-hospital survival. The cumulative survival rate was significantly lower in the hypochloremia group compared with the normochloremia (Log rank *P* = 0.0071).

**Table 2:** Associations of time-varying serum chloride with in-hospital and ICU mortality.

		Univariate		Model I		Model II	
	Serum chloride	HR (95% CI)	*P* value	HR (95% CI)	*P* value	HR (95% CI)	*P* value
ICU mortality	Normochloremia	Reference		Reference		Reference	
	Hypochloremia	1.38 (1.10, 1.73)	0.0048	1.40 (1.11, 1.75)	0.0038	1.37 (1.05, 1.80)	0.0187
	Hyperchloremia	0.87 (0.74, 1.04)	0.1241	0.86 (0.73, 1.02)	0.0876	0.87 (0.72, 1.06)	0.1712
In-hospital mortality	Normochloremia	Reference		Reference		Reference	
	Hypochloremia	1.32 (1.10, 1.58)	0.0033	1.37 (1.14, 1.64)	0.0008	1.46 (1.20, 1.80)	0.0003
	Hyperchloremia	0.99 (0.86, 1.14)	0.8862	0.95 (0.83, 1.09)	0.4591	0.89 (0.76, 1.04)	0.1438

Non-adjusted model adjusted for: None.

Adjust I model adjusted for: age; gender; ethnicity.

Adjust II model adjusted for: age; gender; ethnicity; admission weight, admission height; sepsis; diabetes; pneumonia; heart failure; coronary artery disease; hypertension; chronic kidney disease; sodium; potassium; magnesium; calcium; phosphate; bicarbonate; creatinine; blood urea nitrogen; albumin; diuretic; antibiotics; vasopressor; mechanical ventilation.

Abbreviations: CI, confidence interval; HR, hazard ratio; ICU, intensive care units.

### Subgroup analyses

We performed subgroup analyses to assess the relationship between serum Cl and the in-hospital mortality risk (Table 3). Patients were categorized according to age (≤ 62 and > 62 years), sex, sepsis, heart failure, coronary artery disease, hypertension, CKD, diabetes, pneumonia, sodium and bicarbonate levels, the APACHE IV score, AKI stages, and diuretic usage. The association between hypochloremia and outcomes was consistently positive in different subgroups except CKD patients, patients with sodium ≥145 mmol/L, and patients received diuretic. The only significant interaction effects were bicarbonate values for in-hospital mortality (P for interaction = 0.0492) while no interaction effect for any subgroup was observed.

**Table 3:** Subgroup analysis for the effect of serum chloride on in-hospital mortality.

Subgroups	N	Normochloremia Ref	Hypochloremia HR (95% CI)	*P* value	Hyperchloremia HR (95% CI)	*P* value	*P* for interaction
Age (years)							0.6613
< = 62	1607	1	1.23 (0.84, 1.79)	0.2919	0.88 (0.65, 1.19)	0.4082	
>62	2627	1	1.59 (1.26, 2.00)	<0.0001	0.91 (0.75, 1.10)	0.3396	
Gender							0.1403
Male	2414	1	1.37 (1.06, 1.77)	0.0175	0.85 (0.69, 1.05)	0.1399	
Female	1820	1	1.71 (1.27, 2.32)	0.0005	0.92 (0.72, 1.17)	0.4993	
Sepsis							0.2475
Yes	1615	1	1.47 (1.09, 2.00)	0.0123	0.78 (0.61, 0.99)	0.0380	
No	2619	1	1.53 (1.18, 2.00)	0.0016	0.95 (0.77, 1.18)	0.6468	
Heart failure							0.1862
Yes	601	1	1.69 (1.11, 2.56)	0.0140	1.15 (0.74, 1.80)	0.5328	
No	3633	1	1.43 (1.13, 1.81)	0.0028	0.86 (0.72, 1.02)	0.0758	
Hypertension							0.3568
Yes	624	1	1.20 (0.64, 2.23)	0.5710	0.93 (0.59, 1.46)	0.7424	
No	3610	1	1.54 (1.24, 1.91)	0.0001	0.86 (0.73, 1.03)	0.0946	
CAD							0.3813
Yes	217	1	1.36 (0.63, 2.92)	0.4335	0.57 (0.23, 1.30)	0.1793	
No	4017	1	1.49 (1.21, 1.84)	0.0002	0.90 (0.76, 1.06)	0.2053	
CKD							0.3921
Yes	451	1	0.98 (0.49, 1.95)	0.9488	0.94 (0.55, 1.59)	0.8126	
No	3783	1	1.52 (1.22, 1.90)	<0.0001	0.87 (0.74, 1.03)	0.0199	
Diabetes							0.0977
Yes	595	1	1.47 (0.88, 2.47)	0.1415	1.07 (0.66, 1.74)	0.7908	
No	3639	1	1.52 (1.22, 1.89)	0.0002	0.84 (0.71, 1.00)	0.0462	
Pneumonia							0.7757
Yes	784	1	1.57 (0.97, 2.54)	0.0654	0.72 (0.52, 0.98)	0.0388	
No	3450	1	1.45 (1.15, 1.82)	0.0014	0.94 (0.78, 1.13)	0.4873	
AKI stages							0.3447
Stage 1	850	1	1.45 (0.98, 2.15)	0.0659	0.83 (0.60, 1.15)	0.2574	
Stage 2	185	1	2.67 (0.88, 8.11)	0.0823	0.92 (0.39, 2.20)	0.8532	
Stage 3	1149	1	1.36 (1.02, 1.81)	0.0345	0.83 (0.62, 1.10)	0.1914	
Apache IV score							0.2623
<72.5	1714	1	1.43 (0.91, 2.24)	0.1222	1.05 (0.75, 1.48)	0.7777	
≥72.5	2520	1	1.54 (1.24, 1.91)	0.0001	0.79 (0.66, 0.94)	0.0075	
Sodium (mmol/L)							0.3295
<135	1064	1	1.56 (1.16, 2.11)	0.0037	0.87 (0.57, 1.31)	0.4970	
135–145	2595	1	1.53 (1.15, 2.05)	0.0036	0.81 (0.66, 0.99)	0.0376	
≥145	575	1	0.95 (0.42, 2.17)	0.9017	0.90 (0.61, 1.34)	0.6048	
Bicarbonate (mEq/L)							0.0492
<20	1658	1	1.65 (1.26, 2.18)	0.0003	0.75 (0.59, 0.96)	0.0193	
20–30	2340	1	1.29 (0.94, 1.75)	0.1109	0.95 (0.76, 1.18)	0.6539	
≥30	236	1	2.12 (0.91, 4.94)	0.0801	1.92 (0.68, 5.43)	0.2204	
Diuretic							0.5035
Yes	545	1	0.87 (0.50, 1.52)	0.6334	0.77 (0.47, 1.28)	0.3190	
No	3689	1	1.60 (1.28, 2.00)	<0.0001	0.89 (0.75, 1.05)	0.1682	

Abbreviations: AKI, acute kidney injury; APACHE, acute physiology and chronic health evaluation; CAD, coronary artery disease; CI, confidence interval; CKD, chronic kidney disease; HR, hazard ratio.

## Discussion

We found that lower serum Cl levels were associated with increased risk of in-hospital and ICU mortality in critically ill patients with AKI after adjusting for potential confounders and a detailed literature search confirmed that, this is the first study to investigate the association between serum Cl levels and mortality in critically ill patients with AKI.

The leading causes of hypochloremia in critically ill patients are a decreased intake or an increased loss of Cl. For example, hypochloremia may be triggered by loss of gastric fluid via vomiting or gastric drainage, water toxicity, excess infusion of hypotonic solutions, malnutrition, diuretic therapy or adrenal insufficiency, heart failure, or impaired renal Cl reabsorption [27]. Dyschloremia is common in critically ill patients and is associated with poor outcomes [28, 29]. Several studies identified hypochloremia as an independent negative prognostic marker in patients with CKD or chronic heart failure [14, 15, 30–32]. Hypochloremia appeared to be independently associated with an increased risk of AKI. Patients with serum Cl levels ≤ 94 mEq/L had a significantly greater risk of AKI than did patients with Cl levels 100–108 mmol/L (odds ratio [OR] 1.7, 95% CI 1.1–2.6, P = 0.01) [20]. Kee et al. analyzed 483 ICU survivors with severe AKI requiring continuous renal replacement therapy; they found that patients with hypochloremia had a significantly higher risk of incomplete renal recovery than did a normochloremia group (OR 5.12, 95% CI 2.56–10.23, P < 0.001). Hypochloremia was also significantly associated with a higher risk of renal failure (OR 2.74, 95% CI 1.19–6.32, P = 0.02) [33].

We found that hypochloremia was independently prognostic of mortality in critically ill patients with AKI. This relationship has not previously been described; the mechanism is thus unknown. Hypochloremia might be a biomarker of a mortality risk or a direct contributor to the pathology leading to mortality. Serum sodium and Cl levels are presumably very highly correlated. Associations between serum sodium levels and adverse outcomes have been reported in elderly AKI patients, as well as ICU patients with AKI [34, 35]. The serum Cl level might be a surrogate of dysnatremia. However, our multivariate indicated that the prognostic utility of hypochloremia in terms of mortality was independent of the sodium level. In critically ill patients, Cl levels might decrease because of Cl loss in the gastrointestinal tract, excessive diuretic therapy, and malnutrition [7, 36]. AKI patients with hypochloremia likely experience hypovolemia attributable to reduced fluid and nutritional intakes. Because our work was retrospective observational study, we could not confirm any causal relationship between hypochloremia and mortality.

The large sample size (4,233 patients from 208 US hospitals) is a strength of our study. However, our work had several limitations. First, although we highlighted the prognostic utility of the serum Cl level in terms of mortality among AKI patients in ICUs, we could not explain the underlying mechanism. Second, we could not assess the effects of changes in serum Cl levels on prognosis. Third, the eICU database lacks information regarding the SCr levels during the 3 months prior to admission, as well as information regarding major adverse events after discharge. Thus, we could not estimate previous renal function or accurately define the AKI stages. The possible predictive utility of the serum Cl for renal, cerebrovascular and cardiovascular events, as well as its long-term predictive value, was not quantifiable. Fourth, selection bias may have been present, considering the retrospective nature of the work. The confounding factors were not equally distributed among the groups. Although multivariate Cox regression analyses were used to control for potential confounders, high-quality clinical trials are required to strengthen our results.

## Conclusions

Lower serum Cl levels after ICU admission were associated with increased in-hospital and ICU mortality among critically ill patients with AKI. These results should be verified in well-designed prospective trials.
